# Thyroid hormone replacement therapy in dialysis/renal insufficiency patients

**DOI:** 10.3389/fendo.2025.1540802

**Published:** 2025-02-24

**Authors:** Xiaolu Zhao, Fan Liu, Saiya Yuan, Fei Wang, Chunyu Li, Congcong Guo, Junyu Zhao

**Affiliations:** ^1^ School of Clinical Medicine, Shandong Second Medical University, Weifang, China; ^2^ Department of Endocrinology and Metabology, The First Affiliated Hospital of Shandong First Medical University and Shandong Provincial Qianfoshan Hospital, Shandong First Medical University, Shandong Key Laboratory of Rheumatic Disease and Translational Medicine, Shandong Institute of Nephrology, Jinan, China

**Keywords:** dialysis, renal insufficiency, thyroid hormone, replacement therapy, levothyroxine

## Abstract

Dialysis/renal insufficiency patients are often accompanied by hypothyroidism due to renal damage, the mechanisms of which are complex. The use of thyroid hormone replacement therapy in such patients has become an important clinical issue. This article reviews the mechanism of hypothyroidism in dialysis/renal insufficiency patients and describes the importance and precautions of thyroid hormone replacement therapy to provide a reference for clinical diagnosis and treatment.

## 1 Introduction

The kidney functions as both an excretory and a crucial endocrine organ, playing a significant role in the body’s metabolism, degradation, and excretion of thyroid hormones (TH) (1). TH play a critical role in regulating growth and development, metabolism, and the maintenance of renal growth. Several factors, including malnutrition, inflammation, metabolic acidosis, medications, mineral deficiencies (e.g., selenium), and dialysis patterns (such as loss of peritoneal effluent), predispose chronic kidney disease (CKD) patients to low levels of thyroid hormones. Similarly, reduced TH levels can lead to kidney damage through various pathways (2). As an important treatment for the life extension of patients with end-stage renal disease, dialysis efficiently removes accumulated metabolic wastes and excess water from the body through the extracorporeal circulation mechanism, thus alleviating the condition. However, dialysis/renal insufficiency and the patient’s medication may interfere with the metabolism and distribution of thyroid hormones. The purpose of this article is to review the mechanism of hypothyroidism in the context of dialysis/renal insufficiency, the need for thyroid hormone replacement therapy, and the precautions to be taken to provide a reference for clinical practice.

## 2 Interaction between dialysis/renal insufficiency and hypothyroidism

### 2.1 Mechanisms of hypothyroidism in dialysis/renal insufficiency patients

Dialysis/renal insufficiency patients tend to be comorbid with hypothyroidism, and a cross-sectional study found that the prevalence of hypothyroidism in hemodialysis patients was 28%, of which 57.8% had subclinical hypothyroidism and 42.2% had overt hypothyroidism (3). The mechanisms involved are complex. Uremia is a syndrome triggered by severe impairment of renal function, which can result in impaired clearance of many hormones and metabolites in the body. Electrolyte disturbances, especially hyperphosphatemia and hypocalcemia, which are common in patients with renal insufficiency, also affect thyroid function. Hyperphosphatemia can inhibit the synthesis and secretion of TH, while hypocalcemia may affect the thyroid cell membrane’s ion- channels, thereby affecting TH’s synthesis and release (4). In addition, the kidneys, as the main metabolizing organs of iodine, have impaired iodine excretion when functioning abnormally. Excess iodine can lead to hypothyroidism, while impaired iodine excretion itself can cause further damage to the kidneys (5, 6). Chronic renal insufficiency patients often exhibit significant hypoalbuminemia, which leads to a substantial decrease in the levels of thyroid hormone-binding globulin and thyroid hormone-binding prealbumin. The synthesis of thyroid hormone (T4) and triiodothyronine (T3) is decreased in patients, resulting in hypothyroidism (7). In addition, a decreased activity of 5’-deiodinase is found in patients with chronic renal insufficiency, resulting in a decreased conversion of T4 to T3, and finally leading to a decreased T3 (8). Patients with chronic renal insufficiency are usually accompanied by varying degrees of anemia, impaired synthesis and secretion of TH due to insufficient blood supply to the thyroid gland, as well as decreased iodine intake in patients due to loss of appetite and poor nutritional status, both of which lead to insufficient synthesis of TH (9). Patients with chronic renal insufficiency have increased glomerular permeability and decreased tubular reabsorption. A large amount of thyroid hormone-binding globulin and T4/T3 complexes are excreted in urine, resulting in significant loss of T4 and T3 (10), affecting thyroid function.

**Figure 1:**
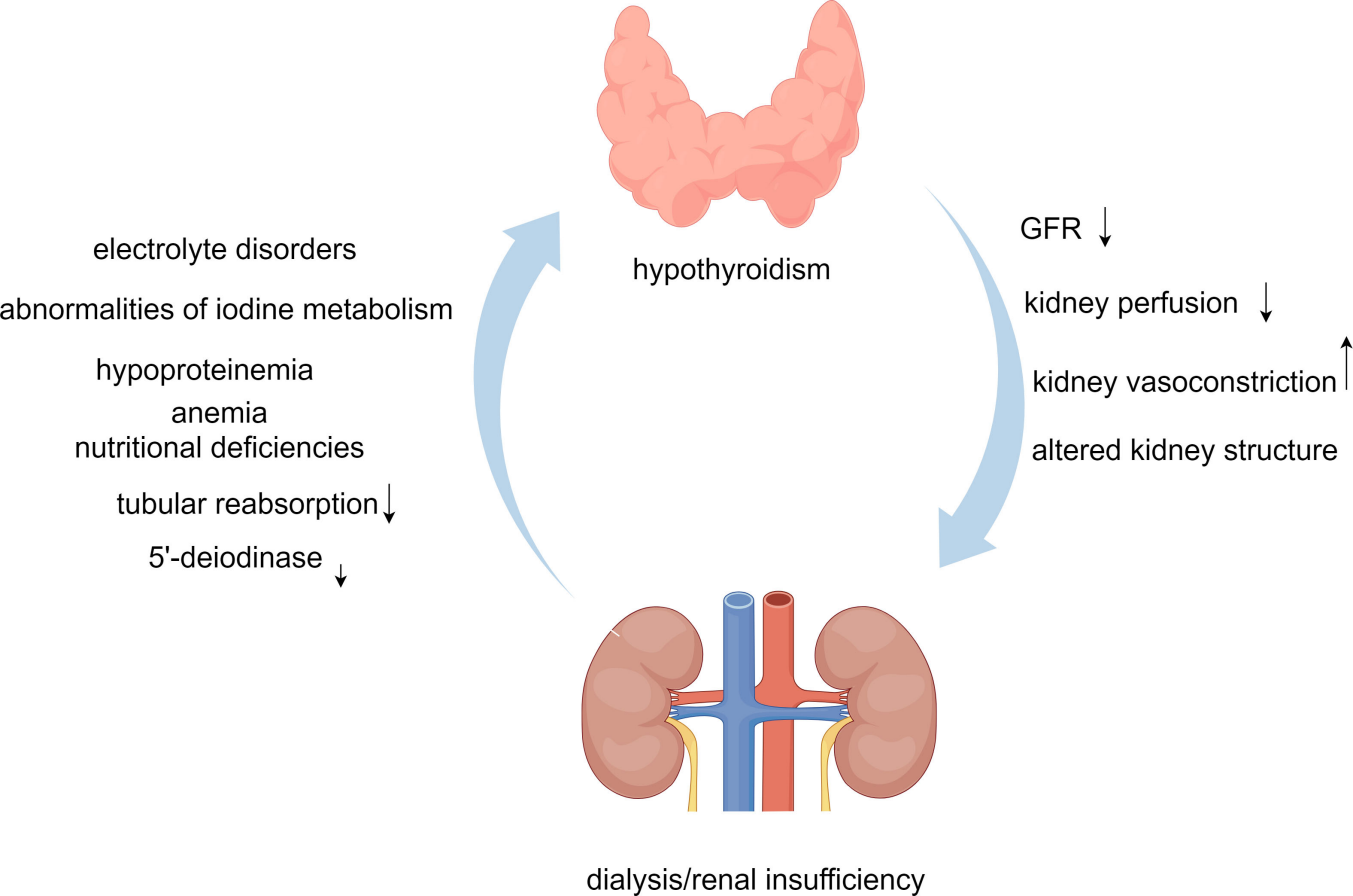
Dialysis/renal insufficiency and hypothyroidism interaction flowchart.

In summary, the mechanisms by which hypothyroidism occurs in patients with renal insufficiency/dialysis are complex and involve electrolyte disorders (e.g., hyperphosphatemia and hypocalcemia), abnormalities of iodine metabolism, hypoproteinemia, and decreased 5’-deiodinase activity, as well as anemia and nutritional deficiencies. In addition, thyroid function is further affected by increased glomerular permeability and decreased tubular reabsorption.

### 2.2 Effects of hypothyroidism on the kidney

The effects of hypothyroidism on the kidneys are manifold. In hypothyroidism, the activity of renal tubular ion transport proteins (e.g., Na+-K+-2Cl- cotransporter) is altered, resulting in increased tubular feedback and ultimately a decrease in GFR (11). A study has reported decreased urine concentrating ability of renal tubules in hypothyroid rats (12). It has also been found that hypothyroidism decreases the synthesis or activity of vasodilators (vascular endothelial growth factor and insulin like growth factor-1), exacerbating renal vasoconstriction, which leads to decreased perfusion to the kidneys (13). It has been directly demonstrated that the expression and secretion of the components of the renin-angiotensin-aldosterone system are directly affected, leading to a decrease in the autoregulation of renal perfusion (14). Reduced reabsorption of sodium, chloride, and water in the proximal tubule and impaired basolateral chloride channels trigger glomerular feedback, resulting in reduced activity of the renin-angiotensin-aldosterone system (15). Animal studies have confirmed that hypothyroidism causes a decrease in GFR, renal plasma perfusion, and renal capillary hydrostatic pressure (16, 17). Hypothyroidism can indirectly lead to decreased renal perfusion by affecting cardiac output and cardiac contractility (18). Hypothyroidism also leads to pathologic changes in the structure of the glomerulus, e.g., thickening of the thylakoid stroma and glomerular basement membrane (19). In addition, the glomerular capillaries’ permeability to proteins increases, leading to the development of large amounts of proteinuria (20).

Thus, renal damage is further exacerbated when patients are in a hypothyroid state. It affects the function and structure of the kidneys by direct or indirect means, resulting in decreased GFR, increased renal vasoconstriction, decreased renal perfusion, increased capillary permeability, and thickening of the tethered matrix and glomerular basement membrane. It even affects renal perfusion by influencing cardiac function. Through the previous discussion, we found that the relationship between dialysis/renal insufficiency and hypothyroidism is complex, and **Figure 1** shows their interaction.

## 3 Importance of thyroid hormone replacement therapy

### 3.1 Slowing down the rate of deterioration of the kidneys

There is a strong link between hypothyroidism and chronic kidney disease (21), and severe hypothyroidism can cause further kidney damage (22). Therefore, thyroid hormone replacement therapy is vital in treating dialysis/renal insufficiency patients. Nie Jingrong et al. (23) showed a specific degree of recovery in renal function after administering a certain dose of levothyroxine (L-T4) in patients with nephrotic syndrome. In addition, animal studies found that the mesangial matrix, mesangial cells, and immune complex precipitation were improved after injection of different doses of T3, and these changes suggest the possibility of TH supplementation in improving renal function (24). In addition, myosin and creatine phosphokinase levels, which are toxic to the renal tubules, decreased in hypothyroidism patients after thyroid hormone replacement therapy (25). Shin D H et al. (26) found in their study that patients with chronic kidney disease undergoing thyroid hormone replacement therapy showed a significant decrease in the slope of glomerular filtration rate (GFR) decline, a lower rate of kidney deterioration, and a delay in achieving end-stage renal disease. Shin D H et al. (27) conducted a study on 309 patients with chronic kidney disease in stages 2-4. After 12 months follow-up, it was found that the GFR decreased in the nontreatment group, and the overall decline rate significantly increased. Kaplan Meier’s analysis also concluded that there was no decrease in renal cell survival. Lu Y and other scholars conducted a case-control study on 90 patients aged 65 and above with subclinical hypothyroidism who were followed up for 36 months. They found that the GFR significantly improved after thyroid hormone replacement therapy (28). Lo JC et al. (29) found a significant increase in GFR before and after thyroid hormone replacement therapy in the same patients by comparing changes in GFR in two populations. Another prospective study treated 30 patients with hypothyroidism with TH therapy. Among them, patients with microalbuminuria and massive proteinuria before treatment showed improvement in proteinuria to normal and microalbuminuria, respectively, and a decrease in serum creatinine after treatment (25). In addition, it was also found that patients with diabetic nephropathy combined with subclinical hypothyroidism had greater oxidative stress, weaker resistance to oxidation, and more severe renal damage, and when L-T4 treatment was given, oxidative stress decreased, and antioxidant levels increased after 24 weeks of follow-up (30). A systematic review also found, by reviewing 12 research papers involving 10,371 patients, that the application of L-T4 reduced the urinary albumin excretion rate, reduced oxidative stress, and provided renal protection compared to placebo (31).

From the above studies, we can find that thyroid hormone replacement therapy for patients on dialysis or with renal insufficiency can improve renal function and slow down the rate of renal deterioration.

### 3.2 Improvement of patients’ quality of life

Dialysis/renal insufficiency patients usually suffer from a sharp decline in quality of life due to malnutrition, cardiovascular complications, and anemia. In previous clinical trials, levothyroxine sodium tablets were found to increase muscle and protein synthesis and improve malnutrition in dialysis patients (32). In addition, Ouyang Yulin and other scholars (33) supplemented 78 patients on maintenance hemodialysis for >6 months with a small dose of L-T4, and a six-month follow-up found that the nutritional status of the patients as well as subjective sensations such as weakness caused by malnutrition improved significantly. Cardiovascular is one of the sensitive target organs of thyroid hormones. Severe hypothyroidism leads to significant abnormalities in myocardial contractility, heart rate, and cardiac electrical conduction (34). The risk of heart failure and coronary heart disease events significantly increases, including mortality caused by coronary heart disease when TSH levels exceed 10 mIU/L (35). The longer the duration of dialysis, the more pronounced the decrease in TH levels, along with a significant decrease in left ventricular ejection fraction, cardiac index, and cardiac output (36). With the addition of L-T4 to dialysis therapy, calcium ATP activity in the myocardial cell membrane and sarcoplasmic reticulum was effectively improved, myocardial con-tractility was enhanced, and cardiac output and coronary blood flow were increased, leading to an improvement in cardiac function (37). Thyroid hormone replacement therapy was found to prevent myocardial fibrosis and cardiomyocyte injury and improve left ventricular diastolic function in relevant animal models (38). 18 elderly patients with chronic heart failure and subclinical hypothyroidism, oral L-T4 therapy was administered, and a one-year follow-up showed an improvement in cardiac function and a decrease in the incidence of ventricular arrhythmias (39). In addition, thyroxine replacement therapy reduced the incidence of cardiovascular disease in patients with diabetic nephropathy and subclinical hypothyroidism and also significantly reduced the risk of cardiovascular disease (40). L-T4 was given to patients with subclinical hypothyroidism for one month, and a three-month follow-up found that the 6-minute walk test and ventricular ejection fraction improved significantly compared to the control group (41). In addition, low-dose L-T4 significantly improve the anemia in patients with uremia and hypothyroidism, but without significant adverse reactions (42).

Therefore, thyroid hormone replacement therapy for patients with dialysis/renal insufficiency combined with hypothyroidism or subclinical hypothyroidism can improve the nutritional status of the patients, improve cardiac function, ameliorate anemia, thus improve the quality of life.

## 4 Potential risks and precautions of thyroid hormone replacement therapy

Although there are some benefits of thyroid hormone replacement therapy for dialysis/renal insufficiency patients in terms of improving renal function and quality of life, there are still some risks concerned.

### 4.1 Impact of different dialysis modalities

Researches have shown that different dialysis methods have varying effects on thyroid function, and treatment effectiveness can also be improved by assisting thyroid hormone replacement therapy in improving patients’ nutritional status, quality of life, and other aspects. Patients undergoing maintenance hemodialysis often have thyroid dysfunction, mainly manifested as a decrease in FT4 (43). In addition, different calcium ion concentrations of dialysate used during hemodialysis will affect parathyroid hormone levels (44), further affecting thyroid function. Adding methods such as hemoperfusion or hemodiafiltration to hemodialysis can significantly improve the removal of parathyroid hormone, thus positively affecting the function of the thyroid gland (45). The high-flux dialyzer is more thorough in removing toxins, allowing the conversion and secretion of thyroxine to be more consistent with the body’s normal physiology (46). In a clinical study, 82 patients with chronic renal failure were divided into two groups, in which the observation group undergoing high-throughput dialysis was compared with the control group, high-throughput dialysis can better reduce the inflammatory response of patients with chronic renal failure, improve the antioxidant and immune ability, and help to maintain the metabolic balance of calcium and phosphorus (47). However, high-flux dialyzers not only extend the range of diffusible molecules but also extend the possibility of convective mass transfer into the patient’s body due to the reverse osmosis of the dialysate, so when the dialysate of high-flux dialysis is not pure enough, the bacteria in the dialysate enter the patient’s body through reverse transport and exacerbate the patient’s inflammatory response (48). In stable dialysis populations, β-2-microglobulin can be removed more efficiently with the use of ultraflux Helixone membranes, with no negative impact on nutrient parameters or small molecule removal despite increased albumin loss (49). Patients treated with online hemodialysis filtration maintain higher levels of antioxidant defenses, which may balance the high oxidative stress during dialysis (50).

Therefore, when thyroid hormone replacement therapy is performed in dialysis patients, the advantages and disadvantages of various dialysis should be considered comprehensively, and the selection of different dialysis modalities according to the patient’s condition may be more helpful to improve the therapeutic effect.

### 4.2 Cardiovascular risk management

Thyroid hormone replacement therapy in dialysis/renal insufficiency patients may improve cardiac function while also posing a number of potential risks, such as nervousness, palpitations, atrial fibrillation, and worsening angina pector-is (51). Even if a patient does not have significant coronary artery disease, it can cause myocardial ischemia, increased coronary spasm, etc.,when high doses of thyroxine are applied (52). Excessive thyroid hormone can cause myocardial hypertrophy, and its mechanism of action includes the direct action of the hormone on the heart, Ca2+ transport, generation of oxygen free radicals, depletion of ATP, and activation of the renin-angiotensin system (53). Thyroxine affects collagen synthesis and, thus, cardiac remodeling primarily by influencing the activity of fibroblasts and matrix metalloproteinases (54). Patients with end-stage renal disease tend to suffer from left ventricular dysfunction and entricular hypertrophy. Some clinical studies have found that with serum TSH concentrations above 4.0 mU/L, the probability of cardiovascular disease is significantly increased in patients on long-term thyroid hormone replacement therapy, especially in male patients (55). It has been found that short-term supplementation of small doses of thyroxine in patients with end-stage renal disease can improve the function of the left heart, but it can also accelerate the heart rate and increase myocardial oxygen consumption, further aggravating the cardiac disease of patients. Therefore, careful consideration should be given to the dose of thyroxine in patients with end-stage renal disease (56).

Among patients maintained on renal dialysis, the cardiovascular morbidity rate is extremely high, and the hypothyroidism caused by dialysis also affects cardiac function in the adoption of thyroxine replacement therapy for these patients, although it can improve cardiac function, it may also lead to the aggravation of the condition or the emergence of new problems. Therefore, regular monitoring of cardiac function by echocardiogram and other means during thyroid hormone replacement therapy not only reduces the mortality rate of patients but also enables thyroid hormone replacement therapy to exert its best therapeutic effect.

### 4.3 Influence of drug and type of preparation on the absorption of levothyroxine

The pharmacologic effects of the drugs on each other may interfere with the absorption of L-T4 when patients receive multiple medications at the same time and affect the efficacy of treatment. Several studies have shown that there are interactions between L-T4 and other drugs such as protease inhibitors, phenytoin, calcium formulations, proton-pump inhibitors, orlistat, sevelamer, and amiodarone, which affect the absorption of L-T4. **Table 1** summarizes the drugs and mechanisms of action that affect L-T4 uptake (57, 58). L-T4 is mainly absorbed in the jejunum and ileum (59), while proton-pump inhibitors reduce the absorption of L-T4 in the intestine by affecting its acidity and alkalinity (60). One study found that TSH returned to normal one month after discontinuing sevelamer and changing the duration of L-T4 administration but increased after reintroduction of sevelamer (61). Calcium formulations can affect the absorption of L-T4. The impact on the absorption of L-T4 is relatively small, when patients take both L-T4 and prescribed doses of calcium formulations. However, a low L-T4 dose, the absorption of L-T4 decreases by 20% -25% (62). Cholestyramine affects L-T4 absorption across the intestinal wall, and ingestion of cholestyramine and levothyroxine must be separated by 4-5 hours for L-T4 absorption to normalize (63, 64). Colesevelam reduces L-T4 absorption by 96%, and sucralfate and ferrous sulfate have insignificant effects on L-T4 absorption (65). In a report by Madhava and Hardley, a patient who underwent thyroidectomy had a stable serum TSH when taking L-T4 alone, which was markedly elevated with the addition of orlistat, after discontinuing orlistat and increasing the dose of L-T4, TSH was again suppressed (66). Amiodarone is not only a structural analog of TH but is also capable of destroying thyroid follicles (67). A clinical study found that serum TSH levels were significantly elevated in TH-treated patients given aluminum hydroxide preparations for 2-4 weeks, suggesting that aluminum hydroxide reduces the bioavailability of L-T4 either by nonspecific adsorption or by forming a complex with L-T4 (68). Postoperative imatinib administration in patients with thyroidectomized thyroids resulted in a significant decrease in serum TSH, and an increase in the amount of L-T4 treatment was required compared with the previous (69). The enzyme inducer carbamazepine enhances TH metabolism through enzyme induction (70). Clinical treatment with carbamazepine in nine patients with hypothyroidism showed a decrease in serum TH levels and no significant change in TSH levels (71).

**Table 1:** The interaction between drugs and L-T4.

mechanism of action	drugs
Decrease T4 absorption	Bile Acid Sequestrants、 Antacids、PPI、Orlistat,etc.
Increased degradation of L-T4	Phenobarbital、Rifampin
Inhibition of L-T4 binding	Salicylates、Carbamazepine,etc.
nhibition of L-T4 metabolism	Glucocorticoids、Beta-adrenergic antagonists (Propranolol)、Amiodarone etc.
Increase serum thyroxine-binding globulin (TBG) concentration.	Clofibrate、Estrogen-containing /contraceptives、Heroin / Methadone、5-Fluorouracil、Mitotane、 Tamoxifen etc.
Decrease serum thyroxine-binding globulin (TBG) concentration.	Androgens / Anabolic Steroids、Asparaginase、 Glucocorticoids、Slow-Release Nicotinic Acid etc.

The form of the L-T4 preparation also affects absorption. The most common form of L-T4 is the tablet form, which requires an acidic gastric pH after ingestion to be absorbed, with approximately 70% being absorbed when no other factors are altered (72). Therefore, tablets are susceptible to changes in gastric pH. When L-T4 malabsorption occurs in hypothyroid patients, new capsule or liquid formulations can improve drug absorption and maintain TSH levels (73). Even the newer formulations are able to maintain normal TSH values in hypothyroid patients more effectively than tablets for those with or without malabsorption (74). Liquid formulations of L-T4 consist of L-T4 dissolved in liquid glycerin and ethanol, while softgel formulations consist of liquid L-T4 encapsulated in an outer gelatin shell (75, 76). Negro et al. conducted a clinical trial and found that there were more patients with abnormal TSH levels in the tablet group than in the liquid formulation group, and that patients in the tablet group were taking higher doses (77). Cappelli et al. found no statistically significant difference in TSH or TH levels whether liquid L-T4 was taken at breakfast or 30 minutes before breakfast (78). In a prospective cohort study of 24 patients with L-T4 malabsorption due to PPI administration, Vita et al. showed a decrease in serum TSH after replacing tablets with the same dose of liquid formulation (79). A clinical study analyzing patients with impaired gastric acid secretion suggested that for such patients the therapeutic goal could be maintained with a capsule formulation at a lower dose than the tablet L-T4 (80).

L-T4 is the main drug for the treatment of hypothyroidism, and its efficacy will not only be affected by factors such as dosage but also by the influence of other drugs and types of preparations, leading to a decrease in the efficacy of L-T4, resulting in poor therapeutic effect. Therefore, when taking L-T4, the matching of drugs should be fully considered to reduce the adverse effects of the above drugs and types of preparations on L-T4 and try to maximize the efficacy of L-T4.

### 4.4 Regular monitor the thyroid morphology and function

During thyroid hormone replacement therapy, monitoring thyroid morphology and function is critical in ensuring the efficacy of treatment and avoiding potential side effects. Hypothyroidism may be exacerbated when thyroid morphology becomes abnormal during the course of treatment. A higher prevalence of nodular goiter in patients with end-stage renal disease was reported (81). Kaptein et al. found that the prevalence of goiter increased with the duration of dialysis (39% of patients on dialysis for less than a year and 50% of patients on dialysis for more than a year) (82). However, a study conducted by Lin C C et al. (83) concluded that there was no significant relationship between nodular goiter and duration of dialysis. Ana et al. (84) found that nearly a quarter of hemodialysis patients have thyroid nodules larger than 10mm. A clinical study of thyroid ultrasound and calculation of thyroid volume in hemodialysis patients found that the mean thyroid volume increased in hemodialysis patients compared to healthy participants (85). Thyroid function not only assesses and predicts prognosis in patients with dialysis/renal insufficiency but also influences the efficacy of thyroid hormone replacement therapy. If the thyroid function is abnormal, it is accompanied by numerous health problems, such as changes in heart structure and function, cardiovascular lesions, malnutrition, inflammation, and even nodules.

Therefore, regular thyroid ultrasound and functional tests are recommended to assess thyroid morphology and function in dialysis/renal insufficiency patients.

### 4.5 Effect of diet on levothyroxine

In clinical work, it was found that the absorption of L-T4 was affected by some dietary habits or foods, such as soy, coffee, dietary fiber and grapefruit juice, when L-T4 was taken in regular or even high doses. The earliest report on the effect of soy on L-T4 absorption was that a patient with goiter was unable to suppress TSH when taking L-T4 at a dose of 0.3mg/day, and in the process of investigating the influencing factors, it was found that the most probable influence was the soy consumed by the patient. After a period of time, it was found that when the dose of L-T4 was reduced to 0.15mg/day, TSH was completely suppressed (86). A clinical study comparing 8 patients with hypothyroidism who consumed soy with 70 patients on a non-soy diet found that the soy-consuming group took longer to reach normal TSH levels (87). Chorazy et al. reported on an infant who was given L-T4 after birth and was also fed soy formula and found elevated TSH levels (88). But currently there are only a few reported cases, and there is still insufficient evidence to prove it. L-T4 can non-specifically adsorb onto dietary fiber, leading to a decrease in its bioavailability (89). In a clinical study, 13 hypothyroid patients were asked to consume dietary fiber while taking L-T4, and after a period of time, 12 of them were found to have elevated serum TSH or increased doses of L-T4 (90). Some studies have found that coffee binds to L-T4 and affects the absorption of L-T4 in the gut (91). A case report showed that serum TSH levels failed to be normalized when coffee and L-T4 were taken at the same time; after correcting this habit, serum TSH levels were normalized (92). The effect of coffee on the extent of L-T4 uptake varies from study to study depending on the variety, concentration, etc (93). Grapefruit juice may slightly L-T4 absorption is prolonged, but seems to have little effect on its bioavailability (94).

From the above studies we found that by avoiding soy and dietary fiber we can improve the therapeutic effect of L-T4 and suppress TSH levels, thus helping to stabilize thyroid function. However, soy and dietary fiber are known to have many benefits for the human body, and we don’t necessarily need to completely stop using them in our daily lives; we can consider improving the therapeutic effect by increasing the dose of L-T4. Avoid taking L-T4 with coffee or grapefruit juice, or use a time interval program. In fact, in our actual clinical work we have found that patients who eat regularly according to their daily habits do not have great changes in their individual TSH levels.

### 4.6 Practical applications

The goal of thyroxine replacement therapy is to restore normal function of the thyroid gland and to eliminate signs and symptoms. Serum TSH is recognized as a reliable, sensitive and reproducible indicator of thyroid status (95). Patients’ replacement therapy TSH should be maintained in the lower half of the normal range (0.5-2.0 mIU/L), and if serum TSH is elevated or suppressed, modest increases or decreases in the L-T4 dose, respectively, will be required; ideally, the dose should not be increased or decreased by more than 12.5 or 25μg at a time (96). L-T4 treatment requires regular monitoring, and it is clear from the previous discussion that over- or under-supplementation can have detrimental effects. Exogenous supplementation of thyroxine to reestablish the balance of the hypothalamic-pituitary-thyroid axis generally takes 4 to 6 weeks, so serum TSH and FT4 are measured at 4- to 6-week intervals during the initial phase of treatment (97). Determine an adequate replacement dose, test TSH again in 6 months, and measure TSH at 12-month intervals thereafter (98).

## 5 Summary

In conclusion, a strong association exists between dialysis/renal insufficiency and hypothyroidism. Dialysis/renal insufficiency can induce hypothyroidism through multiple potential mechanisms. Conversely, hypothyroidism can lead to renal damage in patients with dialysis/renal insufficiency, accelerating renal deterioration and contributing to higher mortality rates in this population. Therefore, thyroid hormone replacement therapy holds significant importance in enhancing renal function and quality of life for patients with dialysis/renal insufficiency. During the replacement therapy, it is recommended to maintain a normal diet, and if the daily diet has soy, coffee and other foods discussed above that affect the absorption of L-T4, a time-interval regimen can be used; avoid taking L-T4 and the above medications at the same time, or liquid or capsule preparations can be chosen. The clearance of L-T4 varies with different dialysis modalities, and a high flux dialyzer can be chosen according to the patient’s actual condition or methods such as hemoperfusion or hemodialysis filtration can be added on top of hemodialysis. TSH in dialysis/renal insufficiency patients should be maintained at 0.5-2.0 mIU/L, and L-T4 should be increased or decreased when it exceeds the upper limit or falls below the lower limit, respectively. TSH should be measured once every 4-6 weeks at the beginning of the treatment period until a final dose is determined, and then again after 6 months of consuming according to the dosage, and then TSH should be measured every 12 months. As research progresses on the metabolism of thyroid hormones and novel drug development, thyroid hormone replacement therapy for patients with dialysis and renal insufficiency is poised to become more precise and efficacious in the future.

## References

[1] Iglesias P Bajo MA Selgas R Díez JJ. Thyroid dysfunction and kidney disease: An update. Rev Endocr Metab Disord. (2017) 18:131–44. doi: 10.1007/s11154-016-9395-7 27864708

[2] Rhee. The interaction between thyroid CM. and kidney disease: an overview of the evidence. Curr Opin Endocrinol Diabetes Obes. (2016) 23:407–15. doi: 10.1097/med.0000000000000275 PMC5094898 27428519

[3] Adani AA Siyad MO Adan AM Jeele MOO. Prevalence and determinants of hypothyroidism in patients on routine hemodialysis in Somalia: A cross-sectional study. Int J Gen Med. (2023) 16:16905–913. doi: 10.2147/ijgm.S403950 PMC10010740 36922965

[4] Li M Zhao QS Xu JM Tang YY Xia N Wing ML. Serum uric acid change and related factors analysis in hypothyroidism patients. Chin J Prev Control Chronic Dis. (2015) 23:646–8. doi: 10.16386/j.cjpccd.issn.1004-6194.2015.09.002

[5] Gong YQ. Effects of uremic toxins in the expression of inflammatory factor and D1 on human hepatocellular carcinoma cells. (2014). Available online at: https://kns.cnki.net/kcms2/article/abstract?v=_mP9DtK6WVfkPU4tUFH2MwFYo2ve3iyZXWsjXOM5-NxGI-rYEZZsGfXpTjLD1PAc-GKeh77tYq5G-Gw1V8OqlKQc9k9KSpsya6-66OVHybiobign1qJzgtOpa9G6qjN3FQGSmI7TNCmKSaytWkcdINA18xd7yQ0VBBpMI-wzNbClhhkYo5XYu2c6Zel9j5vcb0b1ZA9EkXw=&uniplatform=NZKPT&language=CHS.

[6] Yuasa R Muramatsu M Saito A Osuka H Morita T Hamasaki Y. Urinary excretion of thyroid hormone in CKD patients: a proof-of-concept of nephrogenic hypothyroidism. Ren Fail. (2023) 45:2293224. doi: 10.1080/0886022x.2023.2293224 38087476 PMC11001357

[7] Wang S Lin XM Chen SX. The assay of thyroid hormone in uremic patients. Hainan Med J. (2009) 20:191–2. doi: 10.3969/j.issn.1003-6350.2009.05.095

[8] Jiang WY Yu Q Shen QR. Effects of hemodialysis on thyroid hormones in uremic patients. Chin J Integrated Traditional Western Nephrol. (2002) 03:171. doi: 10.3969/j.issn.1009-587X.2002.03.022

[9] Weng GM. Effect of hemodialysis on thyroid hormone levels in patients with chronic renal failure. Chin J Gerontology. (2012) 32:5280–1. doi: 10.3969/j.issn.1005-9202.2012.23.092

[10] Ma EG. Changes and clinical significance of serum thyroid hormones in patients with chronic renal failure. Youjiang Med J. (2008) 04:405–6. doi: 10.3969/j.issn.1003-1383.2008.04.014

[11] Schmitt R Klussmann E Kahl T Ellison DH Bachmann S. Renal expression of sodium transporters and aquaporin-2 in hypothyroid rats. Am J Physiol Renal Physiol. (2003) 284:F1097–104. doi: 10.1152/ajprenal.00368.2002 12569081

[12] Vargas F Moreno JM Rodríguez-Gómez I Wangensteen R Osuna A Alvarez-Guerra M. Vascular and renal function in experimental thyroid disorders. Eur J Endocrinol. (2006) 154:197–212. doi: 10.1530/eje.1.02093 16452532

[13] Schmid C Brändle M Zwimpfer C Zapf J Wiesli P. Effect of thyroxine replacement on creatinine, insulin-like growth factor 1, acid-labile subunit, and vascular endothelial growth factor. Clin Chem. (2004) 50:228–31. doi: 10.1373/clinchem.2003.021022 14709659

[14] Vargas F Rodríguez-Gómez I Vargas-Tendero P Jimenez E Montiel M. The renin-angiotensin system in thyroid disorders and its role in cardiovascular and renal manifestations. J Endocrinol. (2012) 213:25–36. doi: 10.1530/joe-11-0349 22043064

[15] Basu G Mohapatra A. Interactions between thyroid disorders and kidney disease. Indian J Endocrinol Metab. (2012) 16:204–13. doi: 10.4103/2230-8210.93737 PMC3313737 22470856

[16] Bradley SE Stéphan F Coelho JB Réville P. The thyroid and the kidney. Kidney Int. (1974) 6:346–65. doi: 10.1038/ki.1974.119 4431166

[17] Falk SA Buric V Hammond WS Conger JD. Serial glomerular and tubular dynamics in thyroidectomized rats with remnant kidneys. Am J Kidney Dis. (1991) 17:218–27. doi: 10.1016/s0272-6386(12)81132-2 1992665

[18] Klein I Danzi S. Thyroid disease and the heart. Circulation. (2007) 116:1725–35. doi: 10.1161/circulationaha.106.678326 17923583

[19] Spahia N Rroji M Barbullushi and G. Spasovski. Subclinical Hypothyroidism M. Kidney, and heart from normal to uremic milieu. Metab Syndr Relat Disord. (2023) 21:415–25. doi: 10.1089/met.2023.0057 37433213

[20] Wheatley T Edwards OM. Mild hypothyroidism and oedema: evidence for increased capillary permeability to protein. Clin Endocrinol (Oxf). (1983) 18:627–35. doi: 10.1111/j.1365-2265.1983.tb00601.x 6684003

[21] Yuasa R Ohashi Y Saito A Tsuboi K Shishido S Sakai K. Prevalence of hypothyroidism in Japanese chronic kidney disease patients. Ren Fail. (2020) 42:572–9. doi: 10.1080/0886022x.2020.1777162 PMC7946052 32567453

[22] Salomon MI Di Scala V Grishman E Brener J Churg J. Renal lesions in hypothyroidism: a study based on kidney biopsies. Metabolism. (1967) 16:846–52. doi: 10.1016/0026-0495(67)90186-2 6072218

[23] Nie JR Gao QY Cheng XM. Study the serum levels of thyroid hormone on patients with primary nephritic syndrome and the efficacy of thyroid hormone treatment. Chin J Clin Healthcare. (2008) 01:33–4. doi: 10.3969/j.issn.1672-6790.2008.01.015

[24] Wang H Yu JJ Shi XH. Effects of thyroid hormone replacement therapy on renal function and renal endothelial growth factor in rats with nephrotic syndrome. Chin J Integrated Traditional Western Nephrol. (2022) 23:264–265+285. doi: 10.3969/j.issn.1009-587X.2022.03.022

[25] Chou KM Chiu SY Chen CH Yang NI Huang BY Sun CY. Correlation of clinical changes with regard to thyroxine replacement therapy in hypothyroid patients: focusing on the change of renal function. Kidney Blood Press Res. (2011) 34:365–72. doi: 10.1159/000328324 21646817

[26] Shin DH Lee MJ Lee HS Oh HJ Ko KI Kim CH. Thyroid hormone replacement therapy attenuates the decline of renal function in chronic kidney disease patients with subclinical hypothyroidism. Thyroid. (2013) 23:654–61. doi: 10.1089/thy.2012.0475 PMC3675831 23281965

[27] Shin DH Lee MJ Kim SJ Oh HJ Kim HR Han JH. Preservation of renal function by thyroid hormone replacement therapy in chronic kidney disease patients with subclinical hypothyroidism. J Clin Endocrinol Metab. (2012) 97:2732–40. doi: 10.1210/jc.2012-1663 22723335

[28] Lu Y Guo H Liu D Zhao Z. Preservation of renal function by thyroid hormone replacement in elderly persons with subclinical hypothyroidism. Arch Med Sci. (2016) 12:772–7. doi: 10.5114/aoms.2016.60965 PMC4947625 27478458

[29] Lo JC Chertow GM Go AS Hsu CY. Increased prevalence of subclinical and clinical hypothyroidism in persons with chronic kidney disease. Kidney Int. (2005) 67:1047–52. doi: 10.1111/j.1523-1755.2005.00169.x 15698444

[30] Chen Y Wu G Xu M. The effect of L-thyroxine substitution on oxidative stress in early-stage diabetic nephropathy patients with subclinical hypothyroidism: a randomized double-blind and placebo-controlled study. Int Urol Nephrol. (2018) 50:97–103. doi: 10.1007/s11255-017-1756-y 29196928

[31] Manshahia PK Nahar S Kanda S Chatha U Odoma VA Pitliya A. Systematic review to gauge the effect of levothyroxine substitution on progression of diabetic nephropathy in patients with hypothyroidism and type 2 diabetes mellitus. Cureus. (2023) 15:e44729. doi: 10.7759/cureus.44729 37809188 PMC10557367

[32] Liu Q Li CZ Gao ZH. Effects of low-dose levothyroxine sodium tablets on the nutritional status and complications in patients with PEW andlow T3 syndrome receiving mainternance hemodialysis. Hebei Med J. (2018) 40:1701–1703+1706. doi: 10.3969/j.issn.1002-7386.2018.11.025

[33] Ou YYL. Effect of low-dose levothyroxine on the nutritional status of patients on continuous hemodialysis. Jilin Med J. (2012) 33:4971–2. doi: 10.3969/j.issn.1004-0412.2012.23.034

[34] Chojnowski K Bielec A Czarkowski M Dmowska-Chalaba J Kochanowski J Wasowska A. Repeated ventricular. Cardiol J. (2007) 14:198–201. 18651458

[35] Ettleson MD. Cardiovascular outcomes in subclinical thyroid disease: an update. Curr Opin Endocrinol Diabetes Obes. (2023) 30:218–24. doi: 10.1097/med.0000000000000818 PMC10527066 37288727

[36] Tao QL Huang YY. Serum thyroid hormone expression and its relationship with cardiac function in chronic renal failure hemodialysis patients. J Rare Uncommon Dis. (2024) 31:60–1. doi: 10.3969/j.issn.1009-3257.2024.5.025

[37] Chen PL Liu J. Effect of thyroxine on cardiac function in uremic patients. Effect thyroxine cardiac Funct uremic patients. (2017) 37:56–8. doi: 10.19528/j.issn.1003-3548.2017.04.024

[38] Weltman NY Pol CJ Zhang Y Wang Y Koder A Raza S. Long-term physiological T3 supplementation in hypertensive heart disease in rats. Am J Physiol Heart Circ Physiol. (2015) 309:H1059–65. doi: 10.1152/ajpheart.00431.2015 PMC4591362 26254335

[39] Wu JY Yang L Wang L Qiao CD Liu YM Yang JG. Effects of low-dose levothyroxine on heart function in elderly chronic heart failure patients with subclinical hypothyroidism. J Lanzhou Univ (Medical Sciences). (2011) 37:51–4. doi: 10.13885/j.issn.1000-2812.2011.02.007

[40] Seo C Kim S Lee M Cha MU Kim H Park S. Thyroid hormone replacement reduces the risk of cardiovascular diseases in diabetic nephropathy patients with subclinical hypothyroidism. Endocr Pract. (2018) 24:265–72. doi: 10.4158/ep-2017-0017 29547051

[41] Zheng WT. Therapeutic effects of levothyroxine in patients with subclinical hypothyroidism with cardiovascular disease. Xinxueguanbing Fangzhi Zhishi. (2023) 13:9–12.

[42] Liu FY. Observation on the efficacy of small-dose levothyroxine in the treatment of uremia with hypothyroidism. Pract Clin Med. (2009) 10:29–30.

[43] Wang J Xiao B Lin H. Clinical characteristics of thyroid dysfunction in maintenance hemodialysis patients. Chin J Health Care Med. (2019) 21:242–5. doi: 10.3969/issn.1674-3245.2019.03.014

[44] Zuo L Sun LY Wang M. Effects of different calcium concentration dialysate on calcium balance and iPTH during hemodialysis. Chin J Nephrol. (2004) 03:60–3. doi: 10.3760/j.issn:1001-7097.2004.03.016

[45] Chen XM Wang CL Huang ZZ Liu F. Effects of different dialysis modalities on parathyroid hormone clearance in maintenance hemodialysis patients. Clin Medicin. (2007) 04:47–8. doi: 10.3969/j.issn.1003-3548.2007.04.034

[46] Fan WK Liang YY Gao AH Yang H Chen GX Zhou SX. The affect of high flux hemodialysis on serum thyroid hormone. Modern Hosp. (2005) 12:29–31. doi: 10.3969/j.issn.1671-332X.2005.12.012

[47] Li S Li H Wang J Yin L. Impact of high-flux hemodialysis on chronic inflammation, antioxidant capacity, body temperature, and immune function in patients with chronic renal failure. J Healthc Eng. (2022) 2022:20227375006. doi: 10.1155/2022/7375006 PMC8979677 35388330

[48] Schiffl H. High-flux dialyzers, backfiltration, and dialysis fluid quality. Semin Dial. (2011) 24:1–4. doi: 10.1111/j.1525-139X.2010.00786.x 21299628

[49] Pellicano R Polkinghorne KR Kerr PG. Reduction in beta2-microglobulin with super-flux versus high-flux dialysis membranes: results of a 6-week, randomized, double-blind, crossover trial. Am J Kidney Dis. (2008) 52:93–101. doi: 10.1053/j.ajkd.2008.02.296 18423807

[50] Navarro-García JA Rodríguez-Sánchez E Aceves-Ripoll J Abarca-Zabalía J Susmozas-Sánchez A González Lafuente L. Oxidative Status before and after Renal Replacement Therapy: Differences between Conventional High Flux Hemodialysis and on-Line Hemodiafiltration. Nutrients. (2019) 11:2809. doi: 10.3390/nu11112809 31744232 PMC6893513

[51] Villar HC Saconato H Valente O Atallah AN. Thyroid hormone replacement for subclinical hypothyroidism. Cochrane Database Syst Rev. (2007) 2007:Cd003419. doi: 10.1002/14651858.CD003419.pub2 17636722 PMC6610974

[52] Pantos C Mourouzis I Xinaris C Cokkinos DV. Thyroid hormone and myocardial ischaemia. J Steroid Biochem Mol Biol. (2008) 109:314–22. doi: 10.1016/j.jsbmb.2008.03.011 18430565

[53] Gu WL Chen CC. The mechanism of thyroxin induced myocardial hypertrophy and the intervention of Chinese herbs. Lishizhen Med Materia Med Res. (2007) 07:1560–2. doi: 10.3969/j.issn.1008-0805.2007.07.010

[54] Fang JZ Chen RQ. Advances in the pathologic study of the effect of thyroxine on cardiac enlargement. Herald Med. (2006) 06:558–60. doi: 10.3870/j.issn.1004-0781.2006.06.031

[55] Flynn RW Bonellie SR Jung RT MacDonald TM Morris AD Leese GP. Serum thyroid-stimulating hormone concentration and morbidity from cardiovascular disease and fractures in patients on long-term thyroxine therapy. J Clin Endocrinol Metab. (2010) 95:186–93. doi: 10.1210/jc.2009-1625 19906785

[56] Ou YSX Chen Y Huang AL Liang YM Qian SY Long XD. Study on effect and correlation of thyroid hormone level changes in peritoneal dialysis patients by thyroxin tablet. Chin J Biochem Pharmaceutics. (2015) 35:92–94+97. doi: CNKI:SUN:SHYW.0.2016-02-031

[57] Levothyroxine sodium tablet(2024). Available online at: https://dailymed.nlm.nih.gov/dailymed/getFile.cfm?setid=4ddcaec1-a58f-340b-9bc1-bbe048b8c885&type=pdf (Accessed January 30, 2025).

[58] Garber JR Cobin RH Gharib H Hennessey JV Klein I Mechanick JI. Clinical practice guidelines for hypothyroidism in adults: cosponsored by the American Association of Clinical Endocrinologists and the American Thyroid Association. Thyroid. (2012) 22:1200–35. doi: 10.1089/thy.2012.0205 22954017

[59] Lipp HP. Administration and pharmacokinetics of levothyroxine. In: Kahaly GJ, editor. 70 Years of Levothyroxine. Springer, Cham (CH) (2021). p. 13–22. 36315708

[60] Guzman-Prado Y Vita R Samson O. Concomitant use of levothyroxine and proton pump inhibitors in patients with primary hypothyroidism: a systematic review. J Gen Intern Med. (2021) 36:1726–33. doi: 10.1007/s11606-020-06403-y PMC8175524 33469743

[61] Cataldo E Columbano V Nielsen L Gendrot L Covella B Piccoli GB. Phosphate binders as a cause of hypothyroidism in dialysis patients: practical indications from a review of the literature. BMC Nephrol. (2018) 19:155. doi: 10.1186/s12882-018-0947-9 29966512 PMC6027573

[62] Zamfirescu I Carlson HE. Absorption of levothyroxine when coadministered with various calcium formulations. Thyroid. (2011) 21:483–6. doi: 10.1089/thy.2010.0296 PMC3092723 21595516

[63] Phillips WA Schultz JR Stafford WW. Effects of colestipol hydrochloride on drug absorption in the rat. I. Aspirin, L-thyroxine, phenobarbital, cortisone, and sulfadiazine. J Pharm Sci. (1974) 63:1097–103. doi: 10.1002/jps.2600630714 4851736

[64] Northcutt RC Stiel JN Hollifield JW Stant EG Jr. The influence of cholestyramine on thyroxine absorption. Jama. (1969) 208:1857–61. doi: 10.1001/jama.1969.03160100047012 5818830

[65] Liwanpo L Hershman JM. Conditions and drugs interfering with thyroxine absorption. Best Pract Res Clin Endocrinol Metab. (2009) 23:781–92. doi: 10.1016/j.beem.2009.06.006 19942153

[66] Madhava K Hartley A. Hypothyroidism in thyroid carcinoma follow-up: orlistat may inhibit the absorption of thyroxine. Clin Oncol (R Coll Radiol). (2005) 17:492. doi: 10.1016/j.clon.2005.05.001 16149295

[67] Silva JR Guariento ME Fernandes GA Maciel RM Ward LS. Impact of long-term administration of amiodarone on the thyroid function of patients with Chagas' disease. Thyroid. (2004) 14:371–7. doi: 10.1089/105072504774193212 15186615

[68] Liel Y Sperber AD Shany S. Nonspecific intestinal adsorption of levothyroxine by aluminum hydroxide. Am J Med. (1994) 97:363–5. doi: 10.1016/0002-9343(94)90303-4 7942938

[69] de Groot JW Zonnenberg BA Plukker JT van der Graaf WT Links TP. Imatinib induces hypothyroidism in patients receiving levothyroxine. Clin Pharmacol Ther. (2005) 78:433–8. doi: 10.1016/j.clpt.2005.06.010 16198662

[70] Li ZD Shi XJ Wang HT. The drug interactions and mechanism of levothyroxine sodium with other drugs Pharmaceutical Care and Research. Pharm Care Res. (2003) 01:59–61. doi: 10.3969/j.issn.1671-2838.2003.01.019

[71] Aanderud S Myking OL Strandjord RE. The influence of carbamazepine on thyroid hormones and thyroxine binding globulin in hypothyroid patients substituted with thyroxine. Clin Endocrinol (Oxf). (1981) 15:247–52. doi: 10.1111/j.1365-2265.1981.tb00662.x 6796303

[72] Centanni M Gargano L Canettieri G Viceconti N Franchi A Delle Fave G. Thyroxine in goiter, Helicobacter pylori infection, and chronic gastritis. N Engl J Med. (2006) 354:1787–95. doi: 10.1056/NEJMoa043903 16641395

[73] Fallahi P Ferrari SM Ruffilli I Ragusa F Biricotti M Materazzi G. Advancements in the treatment of hypothyroidism with L-T4 liquid formulation or soft gel capsule: an update. Expert Opin Drug Delivery. (2017) 14:647–55. doi: 10.1080/17425247.2016.1227782 27552635

[74] Ferrari SM Ragusa F Elia G Paparo SR Mazzi V Baldini E. Precision medicine in autoimmune thyroiditis and hypothyroidism. Front Pharmacol. (2021) 12:12750380. doi: 10.3389/fphar.2021.750380 PMC8635786 34867359

[75] Vita R Fallahi P Antonelli A Benvenga S. The administration of L-thyroxine as soft gel capsule or liquid solution. Expert Opin Drug Delivery. (2014) 11:1103–11. doi: 10.1517/17425247.2014.918101 24896369

[76] Colucci P D'Angelo P Mautone G Scarsi C Ducharme MP. Pharmacokinetic equivalence of a levothyroxine sodium soft capsule manufactured using the new food and drug administration potency guidelines in healthy volunteers under fasting conditions. Ther Drug Monit. (2011) 33:355–61. doi: 10.1097/FTD.0b013e318217b69f 21516059

[77] Negro R Valcavi R Agrimi D Toulis KA. Levothyroxine liquid solution versus tablet for replacement treatment in hypothyroid patients. Endocr Pract. (2014) 20:901–6. doi: 10.4158/ep13378.Or 24793916

[78] Cappelli C Pirola I Daffini L Formenti A Iacobello C Cristiano A. A double-blind placebo-controlled trial of liquid thyroxine ingested at breakfast: results of the TICO study. Thyroid. (2016) 26:197–202. doi: 10.1089/thy.2015.0422 26586610

[79] Vita R Saraceno G Trimarchi F Benvenga S. Switching levothyroxine from the tablet to the oral solution formulation corrects the impaired absorption of levothyroxine induced by proton-pump inhibitors. J Clin Endocrinol Metab. (2014) 99:4481–6. doi: 10.1210/jc.2014-2684 25259910

[80] Santaguida MG Virili C Del Duca SC Cellini M Gatto I Brusca N. Thyroxine softgel capsule in patients with gastric-related T4 malabsorption. Endocrine. (2015) 49:51–7. doi: 10.1007/s12020-014-0476-7 25595886

[81] Pakfetrat M Dabbaghmanesh MH Karimi Z Rasekhi A Malekmakan L Hossein Nikoo M. Prevalence of hypothyroidism and thyroid nodule in chronic hemodialysis Iranian patients. Hemodial Int. (2017) 21:84–9. doi: 10.1111/hdi.12453 27364542

[82] Kaptein EM. Thyroid hormone metabolism and thyroid diseases in chronic renal failure. Endocr Rev. (1996) 17:45–63. doi: 10.1210/edrv-17-1-45 8641223

[83] Lin CC Chen TW Ng YY Chou YH Yang WC. Thyroid dysfunction and nodular goiter in hemodialysis and peritoneal dialysis patients. Perit Dial Int. (1998) 18:516–21. doi: 10.1177/089686089801800510 9848631

[84] Da Costa AB Pellizzari C Carvalho GA Sant'Anna BC Montenegro RL Zammar Filho RG. High prevalence of subclinical hypothyroidism and nodular thyroid disease in patients on hemodialysis. Hemodial Int. (2016) 20:31–7. doi: 10.1111/hdi.12339 26246426

[85] Jusufovic S Hodzic E. Role of chronic hemodialysis in thyroid gland morphology disorders. Med Arh. (2011) 65:327–9. doi: 10.5455/medarh.2011.65.327-329 22299290

[86] Bell DS Ovalle F. Use of soy protein supplement and resultant need for increased dose of levothyroxine. Endocr Pract. (2001) 7:193–4. doi: 10.4158/ep.7.3.193 11421567

[87] Conrad SC Chiu H Silverman BL. Soy formula complicates management of congenital hypothyroidism. Arch Dis Child. (2004) 89:37–40. doi: 10.1136/adc.2002.009365 14709499 PMC1755887

[88] Chorazy PA Himelhoch S Hopwood NJ Greger NG Postellon DC. Persistent hypothyroidism in an infant receiving a soy formula: case report and review of the literature. Pediatrics. (1995) 96:148–50. 7596704

[89] Wiesner A Gajewska D Paśko P. Levothyroxine interactions with food and dietary supplements-A systematic review. Pharm (Basel). (2021) 14:206. doi: 10.3390/ph14030206 PMC8002057 33801406

[90] Liel Y Harman-Boehm I Shany S. Evidence for a clinically important adverse effect of fiber-enriched diet on the bioavailability of levothyroxine in adult hypothyroid patients. J Clin Endocrinol Metab. (1996) 81:857–9. doi: 10.1210/jcem.81.2.8636317 8636317

[91] Sharif K Watad A Bragazzi NL Adawi M Amital H Shoenfeld Y. Coffee and autoimmunity: More than a mere hot beverage! Autoimmun Rev. (2017) 16:712–21. doi: 10.1016/j.autrev.2017.05.007 28479483

[92] Benvenga S Bartolone L Pappalardo MA Russo A Lapa D Giorgianni G. Altered intestinal absorption of L-thyroxine caused by coffee. Thyroid. (2008) 18:293–301. doi: 10.1089/thy.2007.0222 18341376

[93] Vita R Saraceno G Trimarchi F Benvenga S. A novel formulation of L-thyroxine (L-T4) reduces the problem of L-T4 malabsorption by coffee observed with traditional tablet formulations. Endocrine. (2013) 43:154–60. doi: 10.1007/s12020-012-9772-2 22932947

[94] Lilja JJ Laitinen K Neuvonen PJ. Effects of grapefruit juice on the absorption of levothyroxine. Br J Clin Pharmacol. (2005) 60:337–41. doi: 10.1111/j.1365-2125.2005.02433.x PMC1884777 16120075

[95] Jonklaas J Bianco AC Bauer AJ Burman KD Cappola AR Celi FS. Guidelines for the treatment of hypothyroidism: prepared by the american thyroid association task force on thyroid hormone replacement. Thyroid. (2014) 24:1670–751. doi: 10.1089/thy.2014.0028 PMC4267409 25266247

[96] Khandelwal D Tandon N. Overt and subclinical hypothyroidism: who to treat and how. Drugs. (2012) 72:17–33. doi: 10.2165/11598070-000000000-00000 22191793

[97] Chinese Society of Endocrinology. Guidelines for diagnosis and management of hypothyroidism in adults. Chin J Of Endocrinol And Metab. (2017) 33:167–80. doi: 10.3760/cma.j.issn.1000-6699.2017.02.018

[98] Garber JR Cobin RH Gharib H Hennessey JV Klein I Mechanick JI. Clinical practice guidelines for hypothyroidism in adults: cosponsored by the American Association of Clinical Endocrinologists and the American Thyroid Association. Endocr Pract. (2012) 18:988–1028. doi: 10.4158/ep12280.Gl 23246686

